# Protective Effect of a Lipid-Based Preparation from *Mycobacterium smegmatis* in a Murine Model of Progressive Pulmonary Tuberculosis

**DOI:** 10.1155/2014/273129

**Published:** 2014-12-07

**Authors:** Maria de los Angeles García, Reinier Borrero, Maria E. Lanio, Yanely Tirado, Nadine Alvarez, Alina Puig, Alicia Aguilar, Liem Canet, Dulce Mata Espinoza, Jorge Barrios Payán, María Elena Sarmiento, Rogelio Hernández-Pando, Mohd-Nor Norazmi, Armando Acosta

**Affiliations:** ^1^Instituto Finlay, 11600 Havana, Cuba; ^2^Center for Protein Studies, Faculty of Biology, Havana University, 10400 Havana, Cuba; ^3^Instituto Nacional de Ciencias Médicas y Nutrición Salvador Zubirán, 14000 México, DF, Mexico; ^4^School of Health Sciences, Universiti Sains Malaysia, Malaysia; ^5^Institute for Research in Molecular Medicine, Universiti Sains Malaysia, 16150 Kubang Kerian, Kelantan, Malaysia

## Abstract

A more effective vaccine against tuberculosis (TB) is urgently needed. Based on its high genetic homology with *Mycobacterium tuberculosis* (Mtb), the nonpathogenic mycobacteria, *Mycobacterium smegmatis* (Ms), could be an attractive source of potential antigens to be included in such a vaccine. We evaluated the capability of lipid-based preparations obtained from Ms to provide a protective response in Balb/c mice after challenge with Mtb H37Rv strain. The intratracheal model of progressive pulmonary TB was used to assess the level of protection in terms of bacterial load as well as the pathological changes in the lungs of immunized Balb/c mice following challenge with Mtb. Mice immunized with the lipid-based preparation from Ms either adjuvanted with Alum (LMs-AL) or nonadjuvanted (LMs) showed significant reductions in bacterial load (*P* < 0.01) compared to the negative control group (animals immunized with phosphate buffered saline (PBS)). Both lipid formulations showed the same level of protection as Bacille Calmette and Guerin (BCG). Regarding the pathologic changes in the lungs, mice immunized with both lipid formulations showed less pneumonic area when compared with the PBS group (*P* < 0.01) and showed similar results compared with the BCG group. These findings suggest the potential of LMs as a promising vaccine candidate against TB.

## 1. Introduction

Bacille Calmette and Guerin (BCG), the only anti-TB vaccine currently available, seems to be only effective against severe childhood forms of the disease but not against adult pulmonary TB and for the control of the transmission. Therefore, there is an urgent need for new improved vaccines against TB [1, 2].

Lipids are considered important molecules involved in the pathogenesis of TB [3–5]. Many of these molecules are localized at the cell surface and are components of the cell wall of mycobacteria [3–5]. The localization of such molecules as well as the fact that they constitute important virulence factors makes them potential valuable targets for the host immune system [3–5]. Although most subunit TB vaccines (with few exceptions) that are in different phases of evaluation are based primarily on proteins [6], we hypothesize that mycobacterial lipid antigens could also be effective vaccine candidates against Mtb infection. In fact, the protective effect against Mtb of a liposome formulation composed of total lipids of Mtb has been reported in the guinea pig model [7]. Liposomes containing lipid fractions of Mtb have been reported to confer specific humoral and cellular immune responses, as well as protection upon Mtb challenge in guinea pigs [8, 9].

Our group has previously reported on the capability of preparations containing chloroform extractable lipids from* Mycobacterium smegmatis* (Ms) to induce specific IgG antibodies as well as a cross-reactive response against a mixture of Mtb antigens in Balb/c mice [10, 11]. Additionally, proteoliposomes from Ms and BCG elicited cross reactive responses against antigenic fractions from Mtb, demonstrating the antigenic similarities between the cell wall components of these non-pathogenic mycobacteria and Mtb [12, 13].

In this study, we evaluated whether a lipid-based preparation from Ms could provide protection against the virulent laboratory Mtb H37Rv strain in Balb/c mice by using the intratracheal progressive pulmonary TB infection model.

In fact, the current study shows that lipid extracts obtained from Ms either adjuvanted with Alum (LMs-AL) or nonadjuvanted (LMs) were able to provide similar protection against Mtb compared to that provided by BCG.

## 2. Materials and Methods

### 2.1. Bacterial Strains

Ms mc^2^155 strain was obtained from the collection of the National Reference Laboratory of Tuberculosis, Pedro Kouri Institute, Cuba. BCG (Phipps) was kindly provided by Marcel A. Behr from the McGill General Hospital, Montreal, Canada.

### 2.2. LMs

Ms was grown in 8% nutrient broth (BIOCEN, Cuba) containing 1% (w/v) yeast extract (Merk, Germany), 0.5% (v/v) glycerol (Riedel de Haen, Germany), and 0.4% (v/v) Tween 80 (Fluka, Germany) for 48 h with continuous agitation (200 rpm) at 37°C. The Mtb H37Rv and BCG Phipps strains were grown to early mid-log phase in Middlebrook 7H9 medium (Difco, Detroit, MI) supplemented with ADC (BBL, Cockeysville, MD) and 0.05% Tween 80 (Sigma Chemical Co., St. Louis, MO) at 37°C with 5% CO_2_ and with continuous agitation. Ziehl-Neelsen staining was performed to determine the presence of mycobacteria in the culture [14]. Lipids from Ms were extracted according to the method of Bligh and Dyer [15] and liposome-like particles were prepared by dehydration and rehydration using the method described previously [16].

### 2.3. Mice

Female Balb/c mice (6–8 weeks) were used for immunization. Mice were maintained in cages fitted with microisolators connected to negative pressure.

### 2.4. Challenge Study

Four groups of mice (*n* = 5 each) were inoculated subcutaneously (100 *μ*L) with either PBS (PBS), BCG (BCG Phipps, 8 × 10^3^ CFU),* LMs* (1 mg of total lipid preparation from Ms), or LMs-AL (1 mg LMs + 1 mg Alum Alhydrogel, Sigma). The animals received two doses of PBS, LMs, and LMs-AL at 0 and 21 days whereas the group immunized with BCG received a single dose on day 0. Two months after the last immunization, all mice were challenged simultaneously by intratracheal exposure to Mtb H37Rv (2.5 × 10^5^ CFU) as described [17, 18]. All procedures were performed in a laminar flow cabinet in a biosafety level III facility.

### 2.5. Bacilli Load

Two months after challenge, the mice were euthanized and one lung from each animal was aseptically removed and homogenized with a Polytron in sterile isotonic saline solution containing 0.05% Tween 80 (Sigma). Homogenized lungs were serially diluted and plated in duplicates on 7H10 agar (Difco Lab cod) plates. The plates were incubated for 3 weeks at 37°C and colonies were counted to determine the total CFU in lungs.

### 2.6. Histopathology and Morphometric Studies

Lung samples (the other lung from each infected Balb/c mouse) were dehydrated and embedded in paraffin. The lung tissues were sectioned at 5 *μ*m thick and stained with haematoxylin and eosin [18]. The areas affected by pneumonia which correspond to alveolar spaces occupied by inflammatory cells were measured and analyzed using Leica Q-win system software (Leica Microsystems Imaging Solutions ltd., Cambridge, UK, 25X). First, the whole area of the lung was measured. Then the areas affected by pneumonia were determined, and the percentage of lung surface affected by pneumonia was calculated in each mouse from the different groups.

### 2.7. Data Management

Measurements were made blind, and data of log_10_ CFU and percentage of pneumonic area/lung are expressed as the mean ± SD. One way ANOVA and Multiple Range tests were used for the determination of significant differences between the groups.

All the procedures were carried out according to the guidelines [19] and approved by the Ethical Committee for Experimentation in Animals of the National Institute of Medical Sciences and Nutrition “Salvador Zubirán” (INCMNSZ) of Mexico.

## 3. Results and Discussion

In the present study, the protective capability against TB in mice of a lipid preparation from Ms (LMs-AL and LMs) using PBS as negative control and BCG as the gold standard was evaluated.

**Figure 1:**
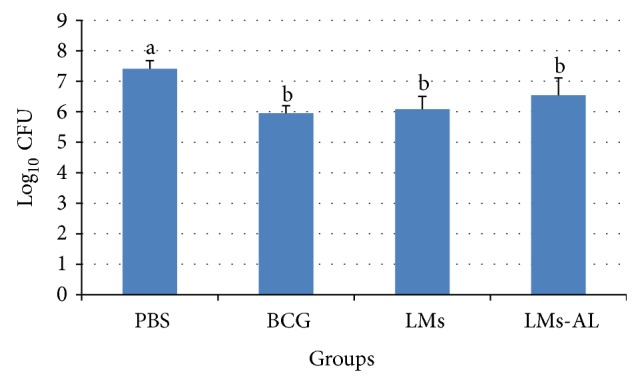
Bacterial load per lung of mice challenged with Mtb H37Rv two months after inoculation. PBS (PBS), BCG (BCG Phipps), LMs (LMs), and LMs-AL (LMs adjuvanted with Alum). Results are presented as mean ± SD. One-way ANOVA and Multiple Range tests were used to analyze the data at *P* < 0.01 level. Different letters denote significant statistical differences among the groups.

As shown in Figure 1, mice immunized with LMs-AL and LMs had significantly reduced bacterial load in the lungs compared to those immunized with PBS (*P* < 0.01). LMs-immunized mice, with and without Alum, showed similar levels of lung CFU compared with each other and with BCG-vaccinated animals (Figure 1).

We have previously shown that mice immunized with LMs produced a significantly higher level of specific IgG response compared to animals receiving PBS alone [10]. We also demonstrated that immunization with this formulation elicited a cross-reactive IgG response against a cell wall fraction from Mtb [10]. Animals receiving the same formulation in combination with Alum or Montanide did not show specific or cross-reactive immune responses [10]. A higher reactivity of IgG antibodies against this formulation was detected in patients with pulmonary TB, compared with healthy individuals, which suggests the expression of similar or cross-reactive epitopes during the active infection with Mtb in humans [10]. When the total lipid extract of Ms was formulated in liposomes containing commercial lipids without adjuvants, a similar induction of specific and cross-reactive IgG response against Mtb was obtained [11].

**Figure 2:**
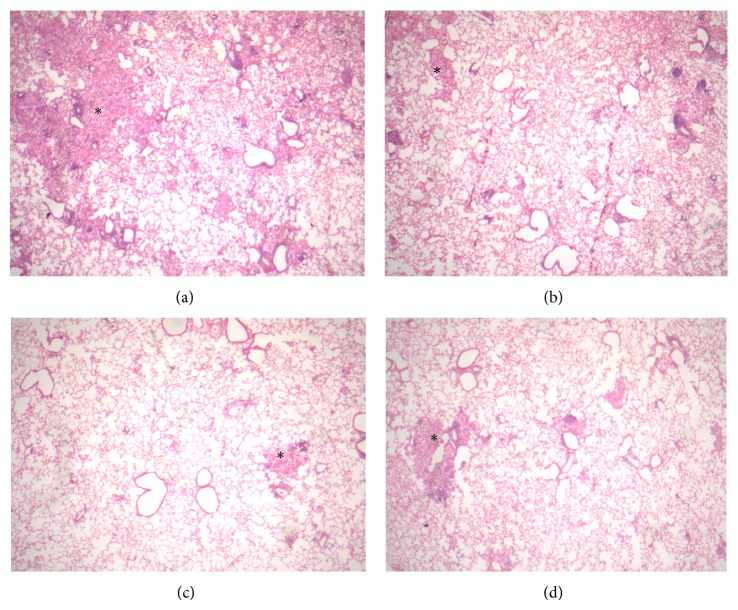
Representative histological micrographs of the lungs of mice. (a) Large areas of inflammatory consolidation or pneumonia (asterisk) in control nonvaccinated mouse. In comparison lesser pneumonia patches (asterisks) are seen in mice vaccinated with BCG (b), LMs (c), or LMs-AL (d). All micrographs H/E staining, 25x magnification.

The histologic study showed several patches of pneumonia in the lungs of control nonvaccinated mice, while vaccinated animals with BCG, LMs, or LMs-AL showed smaller pneumonic areas (Figure 2).

**Figure 3:**
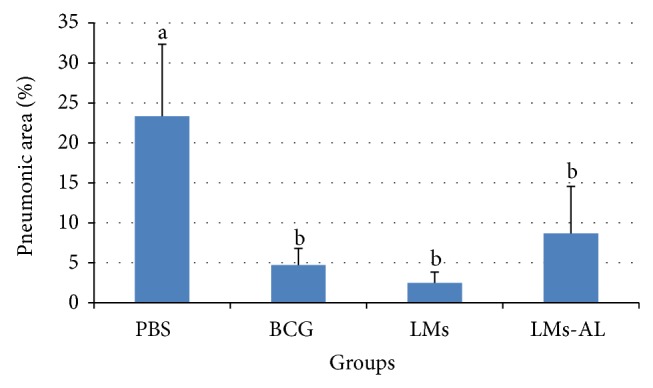
Area of pneumonia in the lungs of mice infected with Mtb H37Rv after two months of inoculation. PBS (PBS), BCG (BCG Phipps), LMs (lipid extract from Ms), and LMs-AL (LMs adjuvanted with alum). The morphometric study was carried out with light microscopy using Leica Q-win System Software. Results are presented as mean ± SD. One-way ANOVA and Multiple Range tests were used to analyze the data. Different letters denote significant statistical differences between the groups (*P* < 0.01).

These histological differences were confirmed by automated morphometry. As shown in Figure 3, animals immunized with LMs and LMs-AL have significantly lower percentage of lung pneumonic area compared to animals immunized with PBS (*P* < 0.01) and no differences with the group immunized with BCG. A previous study has shown similar results using lipids from Mtb in the experimental guinea pig model of TB [7].

Mycobacterial lipids are recognized as antigens with the capacity to stimulate specific T cell responses and play a role in protection against TB, mainly mediated by presentation in the context of CD1 molecules [5, 20–24].

The CD1 molecules have a restricted presence in mice, represented only by CD1d. Therefore, the mechanisms of induction of protection mediated by lipids in this species may differ compared to humans [5, 20].

Regarding the mechanisms of protection mediated by CD1d in mice, it has been demonstrated that the recognition of lipid antigens mediated by CD1d is related to a subpopulation of cells defined as NKT cells which has the capacity to recognize different lipids including self-determinants and exogenous lipids from different microorganisms as* Streptococcus pneumoniae*,* Helicobacter pylori*,* Borrelia burgdorferi*,* Sphingomonas* spp, and Group B* Streptococcus* [25]. Regarding mycobacteria, the presentation mediated by CD1d in mice and humans of phosphatidylinositol mannoside (PIM) which induce the production of gamma interferon and cell-mediated cytotoxic responses in NKT cells [26] has been described. This kind of response could be involved in the protection induced by our vaccine candidate. In our case, the protective effect induced by the immunization with the lipid formulations at the cellular level could be explained by the presentation of different components via CD1d and other unknown mechanisms.

Taking into consideration the induction of specific IgG antibodies and the elicitation of cross-reactive immune responses against Mtb after immunization with formulations containing lipids of Ms in mice [10, 11], the role of specific antibodies in the protection induced by these formulations cannot be ruled out. The contribution of specific antibodies in protection against TB is a subject of debate, but a growing body of evidence supports the potential role of antibodies in the protection against TB [27–36]. The adjuvant effect of a formulation containing total lipid extracts of Ms has been reported [37, 38]. This formulation demonstrated adjuvant effect after the administration with antigens from* Leptospira* and showed stimulatory capacity upon the innate and specific immune responses [37, 38].

Other mycobacterial lipid components such as phosphatidylinositol mannosides have demonstrated mucosal adjuvanticity [39]. The cationic adjuvant formulation AF01 composed of DDA as a delivery vehicle and synthetic mycobacterial cord factor as the immunomodulator has been extensively evaluated [40–45]. This formulation induced superior specific cellular and humoral immune response against ovalbumin compared to other currently used adjuvants [40]. The induced response is independent of the stimulation of toll-like receptors (TLR) 2, 3, 4, and 7 [40]. The use of this adjuvant with Mtb antigens induces strong protection against challenge with Mtb in experimental animals [40, 41, 46]. Other combinations of DDA with lipid fractions of BCG demonstrated high adjuvanticity and the induction of strong protection when combined with Mtb antigens [47, 48].

Considering the intrinsic adjuvant effect of the lipid components of mycobacteria, the protective effect produced by the administration of our lipid formulations could be explained by a combination of nonspecific immunostimulatory effects and the specific stimulation of humoral and cellular immune responses targeting lipid antigens.

The potential of mucosal immunization in the development of new generation vaccine candidates against TB has been recognized [49–51] as well as the role of mycobacterial lipidic components as mucosal adjuvants [39]. These antecedents suggest the possibility of evaluating our experimental vaccine candidate administered by the mucosal route in future studies.

The observation that LMs, either with or without Alum, reduced the bacterial burden as well as pathology in the lungs of mice challenged with Mtb shows the potential of lipids from nonpathogenic mycobacteria as candidate vaccines against the disease.

Compared to previous studies (7–9), the advantage of our study is the use of fast-growing, nonpathogenic mycobacteria, which could facilitate the large-scale use of such a preparation in humans.

## 4. Conclusions

This study is the first to report on the protective efficacy of a preparation based on total lipids extracted from Ms against Mtb infection. LMs and LMs-AL showed better protective efficacy compared to the negative control group and similar protection to the gold standard BCG vaccine in terms of reducing the bacterial load and pneumonic area in the lungs. However, further investigations are necessary to evaluate their eventual potential as improved vaccines against TB.
